# One of Us

**Published:** 1889-02

**Authors:** 


### ONE OF US.

Dr. C. E. Kells' compact arrangement of electrical appliances for office use, including an automatic reel, a tiny incandescent lamp, and other apparatus of his own invention, attracted much attention at the joint meeting at Louisville last summer. His ingenious application of electricity in a series of experiments for testing the conductivity of various filling materials will also be remembered by all who where in attendance at that meeting. It is with pleasure that we note that he has extended the application of the thermostat to a larger and more practical field, in an automatic fire alarm, which has received the indorsementof Prof. Eliha Thompson, and has been indorsed by the boards of underwriters of nine cities; New Orleans allowing a rebate of ten per cent. on all buildings where it is used. It has also been adopted in New York, Cincinnati and Buffalo, and is considered the most reliable system yet invented, as there is no possibility of false alarms, while the ground connection prevents interference by the breakage of overhead wires. The thermostat in his fire alarm is about the size of a watch dial, with the same concave metallic disk and adjustable screw used in the experiments above referred to. The screw is set to operate the transmitting-box at any desired temperature, the thermostat turning in an alarm when that limit is reached. The indicator in the central office shows the street, the number of the building and the story from which the alarm comes. In New Orleans the system is under control of a company officered by the most substantial business men of that city, and of which Dr. Kells is treasurer.

